# Lifetime risks of second primary malignancies after pediatric Hodgkin lymphoma and non-Hodgkin lymphoma

**DOI:** 10.1007/s00432-023-05583-4

**Published:** 2024-01-27

**Authors:** Laduona Wang, Yue Zheng, Ren Luo, Kai Kang, Gabriele Niedermann, Ailin Zhao, Yijun Wu

**Affiliations:** 1 Cancer Center, West China Hospital, Sichuan University, Chengdu, China; 2 Department of Radiation Oncology, Faculty of Medicine, University of Freiburg, Freiburg, Germany; 3 German Cancer Consortium (DKTK), Partner Site Freiburg, Freiburg, Germany; 4 German Cancer Research Center (DKFZ), Heidelberg, Germany; 5 Department of Hematology, West China Hospital, Sichuan University, Chengdu, China

**Keywords:** Pediatric lymphoma, Second primary malignancy, Radiotherapy, Chemotherapy

## Abstract

**Objectives:**

Survivors after pediatric Hodgkin lymphoma (HL) and non-Hodgkin lymphoma (NHL) are with lifetime risk for second primary malignancy (SPM). This necessitates a thorough analysis to better understand the potential long-term health implications for these individuals.

**Methods:**

We used a US-wide population-based cancer registry data to quantify the SPM risk and identify its incidence patterns among pediatric lymphoma patients.

**Results:**

We observed 4.74-fold (95% CI 4.27–5.25) and 3.40-fold (95% CI 2.78–4.10) increased risks of SPM in survivors after pediatric HL and NHL, respectively. Through over 40 years’ follow-up, the cumulative incidence of SPM for pediatric lymphoma was persistently increasing, and here we firstly report the high 40-year cumulative incidence rates of SPM, 22.2% for HL and 12.6% for NHL, suggesting that SPM accounts for a great proportion of deaths among survivors. Of 6805 pediatric lymphomas, 462 (6.36%) developed a SPM, especially second breast and thyroid cancer, followed by hematologic neoplasms including leukemia and NHL. The competing risk analysis demonstrated gender, lymphoma subtype and radiotherapy were significantly associated with SPM. Different risk patterns of SPM were identified between pediatric HL and NHL. Chemotherapy accelerated SPM development but did not increase its incidence risk.

**Conclusion:**

Overall, patients after pediatric lymphoma can be with high lifetime risk of SPM, and more attention should be paid to SPM-related signs for early detection and intervention.

**Supplementary Information:**

The online version contains supplementary material available at 10.1007/s00432-023-05583-4.

## Introduction

With the advent of novel treatments, the life expectancy of lymphoma patients, including pediatric cases, has been significantly extended. However, this progress has given rise to a new and serious concern—the development of second primary malignancies (SPMs). In adult patients, especially those with specific lymphoma subtypes, previous studies have explored the relationships between SPMs and factors such as genetic vulnerability, family history, immunodeficiency, environmental exposure, and various treatment modalities (Chattopadhyay et al. 2020; Chien et al. 2015; Chowdhry et al. 2015; Hemminki et al. 2003; Joelsson et al. 2022; Sud et al. 2017). Yet, there remains a noticeable gap in the research literature when it comes to a comprehensive investigation into pediatric lymphoma survivors, encompassing all subtypes of Hodgkin lymphoma (HL) and non-Hodgkin lymphoma (NHL).

The National Cancer Institute’s Surveillance, Epidemiology, and End Results (SEER) Program, which aggregates data from nearly one-third of the U.S. cancer patient population, provides an invaluable resource for filling this void. Our study, based on SEER database registries, sought to identify patients who received their initial primary lymphoma diagnosis at the age of 19 or younger between 1975 and 2018. We defined SPM as a subsequent malignancy diagnosed at least one year after the initial primary lymphoma diagnosis, excluding individuals who passed away or developed an SPM within the first year following the lymphoma diagnosis.

In examining this research gap and leveraging the extensive SEER database, our study aims to explore the risk factors and incidence rates of SPMs within the unique context of pediatric lymphoma survivors. While acknowledging that previous research has extensively examined these aspects in this population, our study's principal strength lies in the inclusion of a large number of patients, facilitating substantial confirmation of previously identified risk factors. The extended follow-up time further enhances the robustness of our findings. This investigation holds particular relevance due to the distinctive challenges and concerns faced by this specific patient group, necessitating dedicated and comprehensive research. The meticulous definition of SPM in this context, coupled with the exclusion criteria, ensures the precision and relevance of our findings to the pediatric lymphoma survivor population.

## Methods

### Data source

**Fig. 1:**
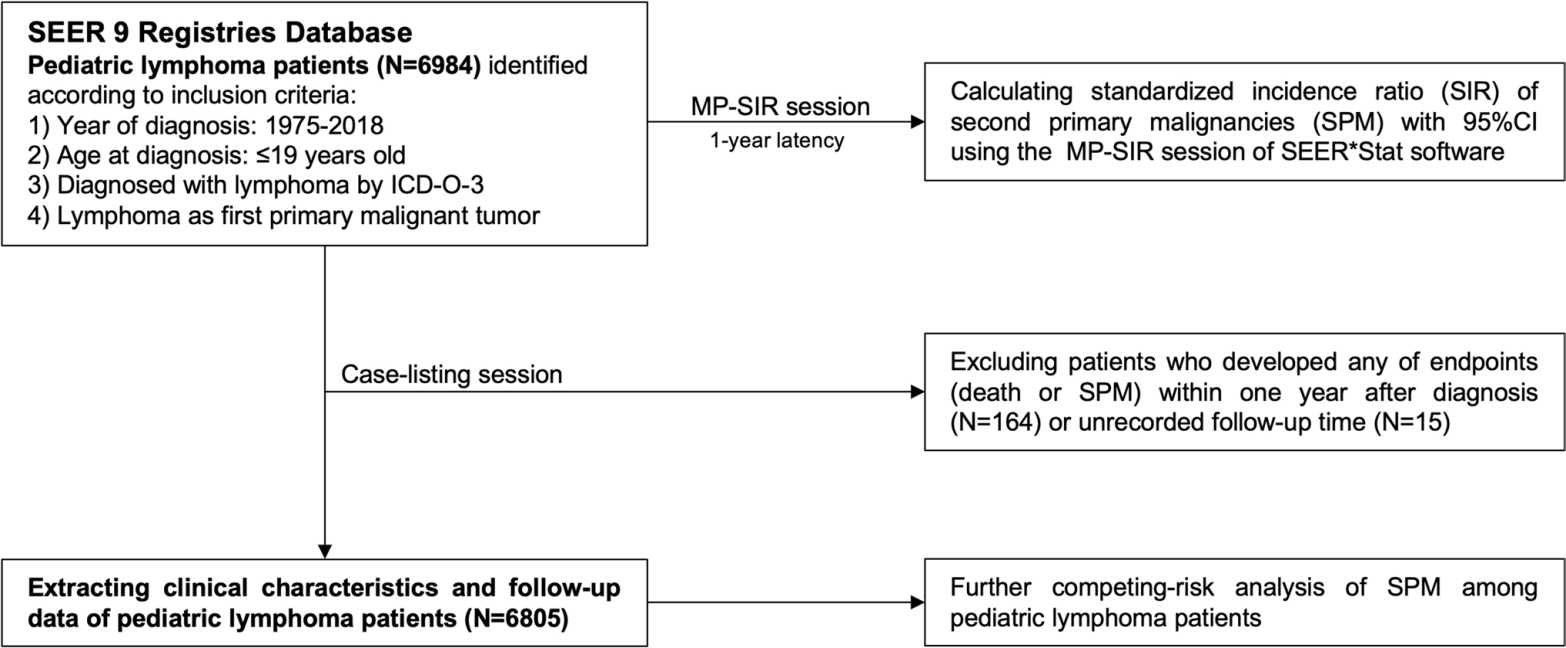
Flowchart of selecting pediatric lymphoma patients

For this research, pediatric lymphoma data spanning the period from 1975 to 2018 was gathered from the SEER database. This valuable resource encompasses information from nine specific U.S. states, all dedicated to combating the growing cancer burden. These contributing states include Connecticut, Michigan, Georgia, California, Hawaii, Iowa, New Mexico, Washington, and Utah. The SEER database, accessible at https://seer.cancer.gov, serves as a comprehensive repository for cancer-related data. Figure 1 provides a visual representation of the study’s workflow and methodology.

### Patient enrollment

Individuals diagnosed with primary lymphoma between the ages of 0–19 years were identified using the 3rd edition of the International Classification of Diseases for Oncology (ICD-O-3). Comprehensive demographic and clinical data were collected from the SEER database, including gender, age at diagnosis, race, latency since diagnosis, primary lymphoma site, lymphoma subtype, Ann Arbor staging, the use of chemotherapy (CT) and radiotherapy (RT), and SPMs. It is essential to emphasize that this study strictly adhered to the Strengthening the Reporting of Observational Studies in Epidemiology guidelines (von Elm et al. 2007).

### Statistical analysis

The standardized incidence ratio (SIR) with a 95% confidence interval (CI) was calculated using the MP-SIR module of the SEERStat software (version 8.3.9) to assess the risk of SPMs among pediatric lymphoma patients. This involved comparing the observed/expected (OE) number of cases. Multivariate competing-risk regression analysis was conducted to identify factors associated with SPM risk. Data were extracted from the pediatric lymphoma cohort (excluding cases with unspecified records) using the Case-Listing module of SEERStat. SPMs and mortality were treated as mutually exclusive competing events. A two-sided P value of less than 0.05 was used to determine statistical significance.

## Results

### Patient characteristics

Between 1975 and 2018, a total of 6984 pediatric patients, aged from 0 to 19 years, were diagnosed with lymphoma (Table 1). The largest proportion of these patients, constituting 54.5% (N = 3812), were in the age group of 15–19 years. Among the diagnosed cases, males accounted for a higher percentage, representing 58.5% (N = 4085), while females made up 41.5% (N = 2899) of the cases. Regarding racial distribution, the majority of patients were White, comprising 81.2% (N = 5669), followed by 11.2% (N = 782) who were Black, and 6.8% (N = 474) from other racial backgrounds. In terms of lymphoma subtypes, 57.0% (N = 3983) were diagnosed with HL, while 43.0% (N = 3001) had NHL. Among the 5,197 cases (74.4%) with available staging information, 19.0% (N = 1329) were classified as stage I, 26.4% (N = 1844) as stage II, 12.1% (N = 842) as stage III, and 16.9% (N = 1182) as stage IV.

**Table 1:** Risks of second primary malignancies (SPM) among pediatric lymphoma patients by demographic characteristics

Characteristic	No. of patients	Observed no. of SPM	Expected no. of SPM	SIR	95%CI	Excess risk (per 10,000)	PY at risk	Mean PY at risk	Mean Age at Exposure	Mean Age at SPM
Total	6984	478	109.7	4.36^b^	3.98–4.77	32.9	112,058	16.0	14.2	35.1
Sex										
Male	4085	172	50.9	3.38^b^	2.89–3.92	18.7	64,953	15.9	13.7	33.9
Female	2899	306	58.8	5.20^b^	4.64–5.82	52.5	47,105	16.3	15.0	35.7
Age at lymphoma diagnosis, years										
< 1	15	1	0.1	19.71	0.26–109.67	63.4	150	10.0	0.6	5.5
1–4	402	12	2.0	5.97^b^	3.08–10.44	17.0	5,891	14.7	3.5	20.4
5–9	984	32	7.5	4.25^b^	2.91–6.00	15.7	15,610	15.9	7.6	27.1
10–14	1771	112	22.9	4.89^b^	4.03–5.89	31.1	28,657	16.2	12.9	33.0
15–19	3812	321	77.2	4.16^b^	3.71–4.64	39.5	61,750	16.2	17.7	37.2
Race										
White	5669	407	99.3	4.10^b^	3.71–4.52	32.3	95,232	16.8	14.4	35.4
Black	782	49	6.7	7.30^b^	5.40–9.65	41.2	10,263	13.1	14.0	34.2
Others^a^	474	22	3.1	7.14^b^	4.48–10.82	32.1	5,890	12.4	13.2	30.5
Unspecified	59	0	NA	NA	NA	NA	NA	NA	NA	NA
Latency since diagnosis, months										
12–59	6984	58	6.2	9.29^b^	7.05–12.01	20.8	24,855	3.6	14.2	18.1
60–119	5628	48	9.9	4.83^b^	3.56–6.40	15.1	25,264	4.5	14.3	22.7
≥ 120	4492	372	93.5	3.98^b^	3.58–4.40	45.0	61,939	13.8	14.3	39.3
Primary lymphoma site										
Lymph nodes of multiple regions	3088	221	49.3	4.48^b^	3.91–5.11	35.5	48,312	15.7	15.1	35.2
Lymph nodes of head, face & neck	1341	119	26.6	4.48^b^	3.71–5.36	36.5	25,338	18.9	14.0	37.2
Intrathoracic lymph nodes	427	31	7.3	4.26^b^	2.90–6.05	35.6	6,663	15.6	14.8	33.7
Lymph nodes of axilla or arm	103	7	1.8	3.88^b^	1.55–8.00	27.5	1,892	18.4	14.6	34.0
Lymph nodes of inguinal region or leg	121	5	2.1	2.39	0.77–5.58	13.1	2,230	18.4	13.5	38.6
Intra-abdominal lymph nodes	130	4	1.7	2.39	0.64–6.13	10.0	2,329	17.9	11.5	21.2
Others/unspecified	1774	91	NA	NA	NA	NA	NA	NA	NA	NA
Lymphoma subtype										
HL	3983	371	78.2	4.74^b^	4.27–5.25	42.3	69,265	17.4	15.8	36.3
Nodal HL	3935	368	77.5	4.75^b^	4.28–5.26	42.4	68,527	17.4	15.8	36.4
Extra-nodal HL	48	3	0.7	4.31	0.87–12.58	31.2	738	15.4	15.8	31.9
NHL	3001	107	31.5	3.40^b^	2.78–4.10	17.6	42,793	14.3	12.1	30.7
Nodal NHL	2053	74	23.4	3.17^b^	2.49–3.98	16.7	30,263	14.7	12.1	31.7
Extra-nodal NHL	948	33	8.1	4.06^b^	2.79–5.70	19.9	12,530	13.2	12.2	28.4
Ann Arbor stage										
Stage I	1329	72	16.2	4.46^b^	3.49–5.61	25.4	22,029	16.6	13.7	32.9
Stage II	1844	95	22.0	4.32^b^	3.49–5.28	27.0	27,005	14.6	15.3	31.6
Stage III	842	40	9.8	4.10^b^	2.93–5.59	24.9	12,163	14.5	14.3	33.7
Stage IV	1182	44	10.1	4.34^b^	3.15–5.82	21.1	16,043	13.6	12.7	25.2
Unstaged	1787	227	NA	NA	NA	NA	NA	NA	NA	NA
Chemotherapy										
No	1520	197	43.7	4.50^b^	3.90–5.18	46.6	32,899	21.6	15.7	38.9
Yes	5464	281	66.0	4.26^b^	3.78–4.79	27.2	79,159	14.5	13.8	32.4
Radiotherapy										
No	3959	161	45.3	3.56^b^	3.03–4.15	21.0	55,039	13.9	13.5	32.2
Yes	2843	301	60.2	5.00^b^	4.45–5.60	45.0	53,466	18.8	15.2	36.4
Unspecified	182	16	NA	NA	NA	NA	NA	NA	NA	NA

All results in this table were calculated and analyzed by MP-SIR session of the software SEER*Stat with a 1-year latency of SPM after the first primary lymphoma

*SPM*, second primary malignancy. *SIR*, standardized incidence ratio. *PY*, person-year

^a^Others in race corresponds to American Indian/AK Native, Asian/Pacific Islander

^b^Significant difference was observed (P < 0.05)

### Elevated risk of second primary malignancies in patients with pediatric lymphoma

Based on the largest-to-date cohort of pediatric lymphoma, we observed an overall 4.36-fold risk of SPMs after pediatric lymphoma (OE: 478/109.7; SIR: 4.36, 95% CI 3.98–4.77; Table 1). There were increased risks of SPMs across subgroups of sex, age, race, latency since diagnosis, primary lymphoma site, subtype, Ann Arbor stage and treatments. The highest SIR was found within 1–5 years (OE: 58/6.2; SIR: 9.29, 95% CI 7.05–12.01) since lymphoma diagnosis, compared with the SIR between 5 and 10 years (OE: 48/9.9; SIR: 4.83, 95% CI 3.56–6.40) or beyond 10 years (OE: 372/93.5; SIR: 3.98, 95% CI 3.58–4.40). Several histological subtypes of lymphoma were identified with notably elevated risks of SPMs (Supplemental Table 1), including nodular lymphocyte-predominant HL (OE: 11/0.9; SIR: 13.00, 95% CI 6.48–23.27), B-cell precursor NHL (OE: 2/0.2; SIR: 11.17, 95% CI 1.25–40.32), T-cell precursor NHL (OE: 3/0.3; SIR: 9.10, 95% CI 1.83–26.59) and mycosis fungoides (OE: 5/0.6; SIR: 9.08, 95% CI 2.93–21.19).

**Fig. 2:**
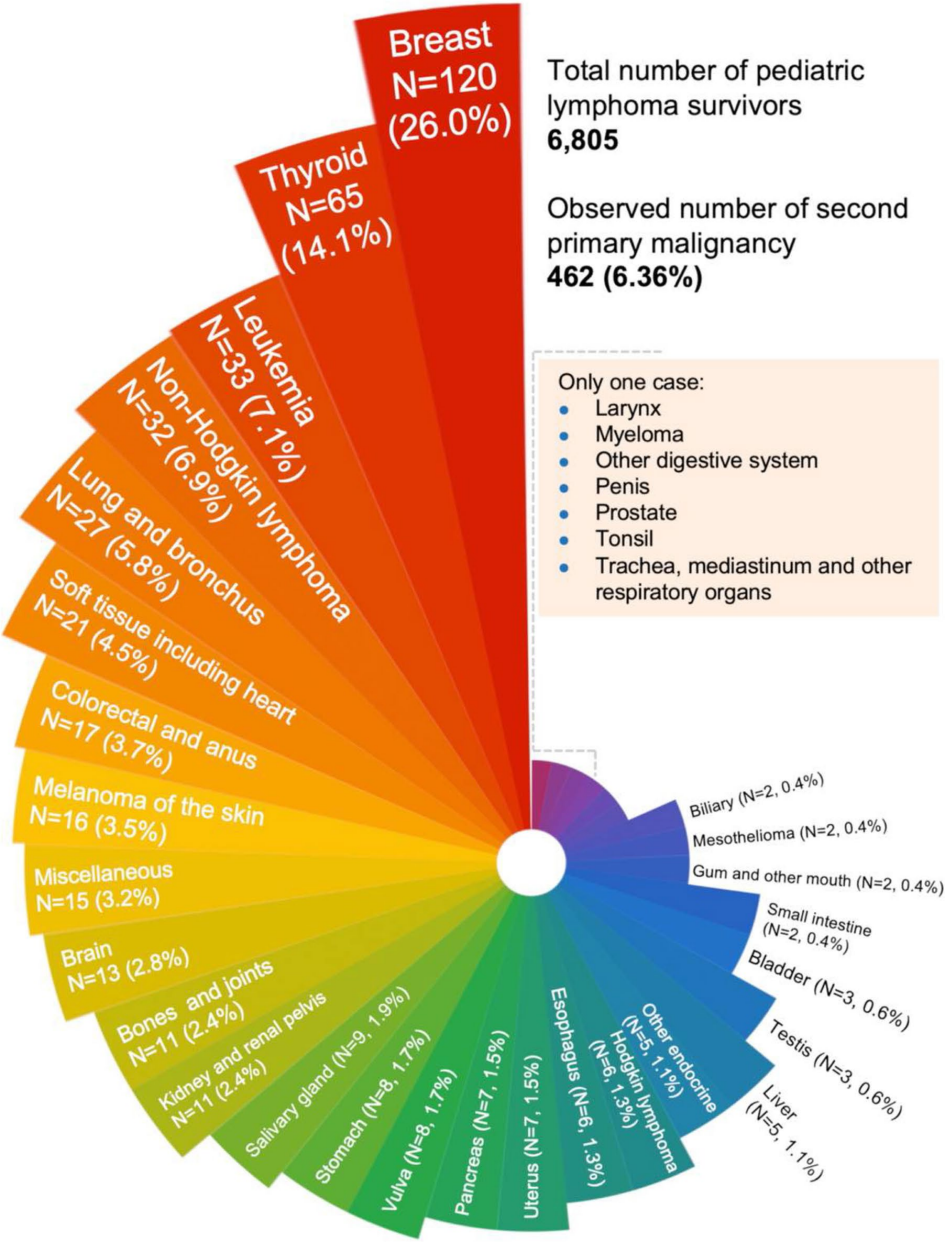
Observed number and percentage of second primary malignancies (SPM) in pediatric lymphoma patients

**Fig. 3:**
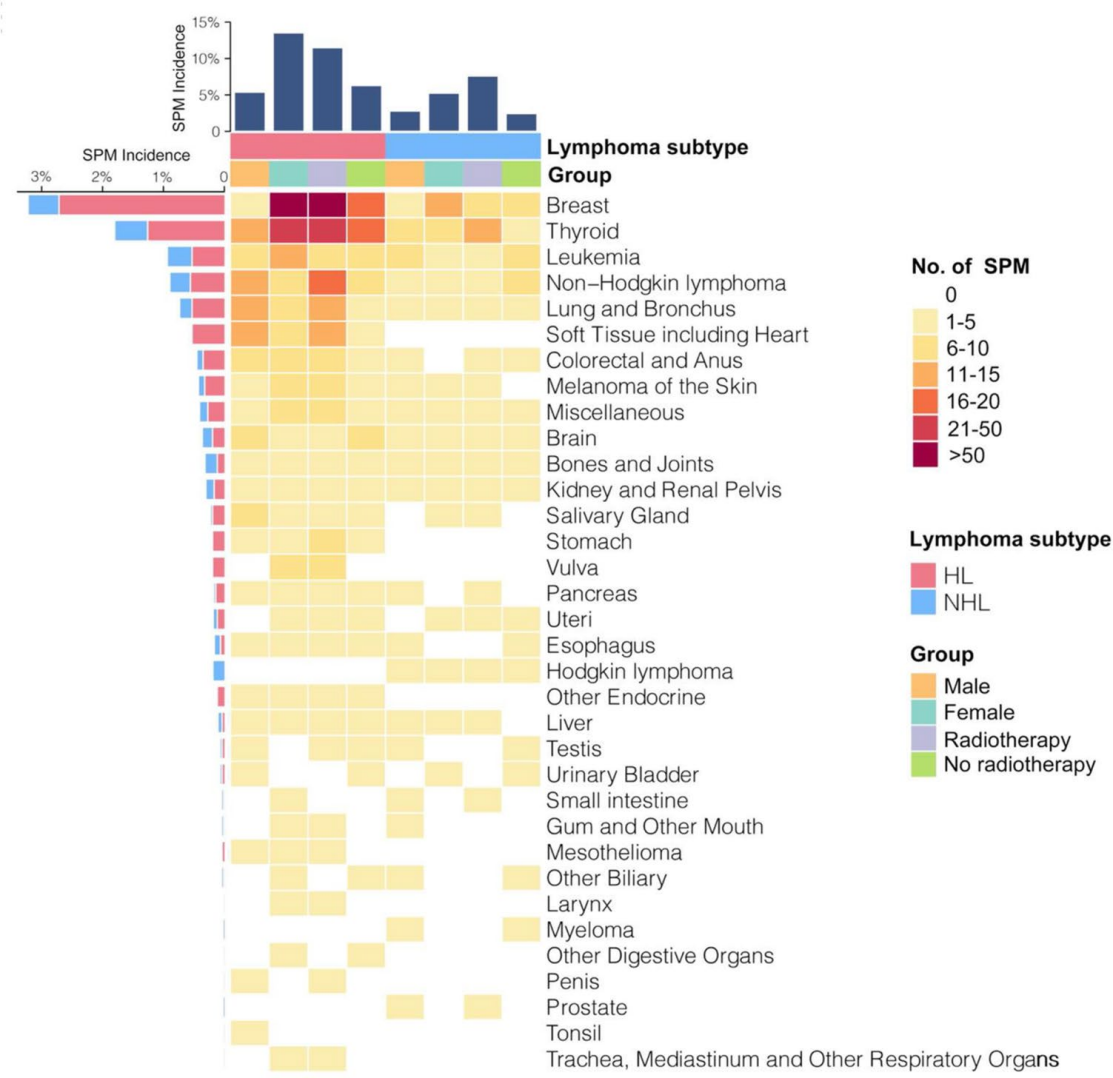
Volcano plot of second primary malignancies (SPM) in pediatric lymphoma patients

**Fig. 4:**
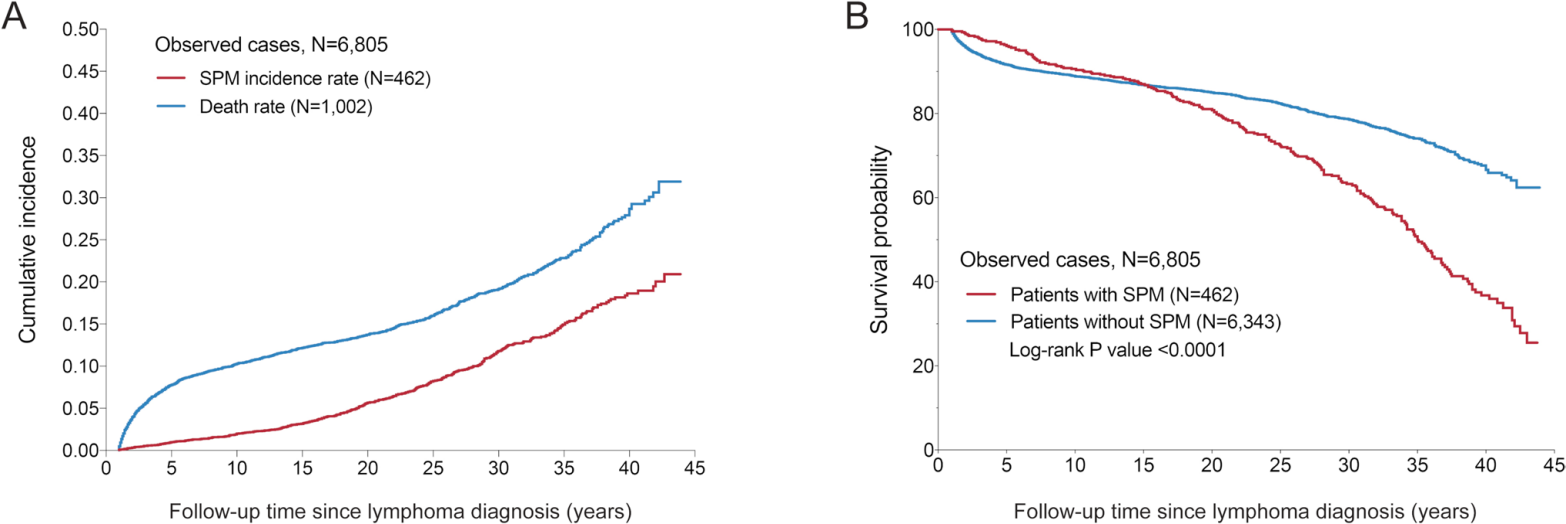
Clinical outcomes of second primary malignancies (SPM) among pediatric lymphoma patients. **A** Cumulative incidence of SPM and death in pediatric lymphoma patients. **B** Kaplan–Meier survival curve for comparing survival between pediatric lymphoma patients with and without SPM

Of 6805 pediatric lymphoma survivors with definitive records, 462 (6.36%) developed SPMs with a median observation time of 15.1 (quartile: 6.5–25.4) years after lymphoma diagnosis. Breast and thyroid cancers, accounting for 26.0% (120/462) and 14.1% (65/462) of all SPM cases, respectively, were the most commonly diagnosed malignancies after both pediatric HL (N = 3849) and NHL (N = 2956), followed by leukemia, NHL, lung cancer, and other entities (Figs. 2, 3). The median interval between lymphoma diagnosis and SPM occurrence was 18.9 (quartile: 10.0–26.6) years. Cumulative incidence rates of SPM were 1.9% at 10 years, 5.6% at 20 years and 18.6% at 40 years, while cumulative death rates were 10.2% at 10 years, 13.7% at 20 years and 27.9% at 40 years (Fig. 4A). Furthermore, the cumulative incidence of SPMs did not display any plateau across over 40 years, which had been rising persistently. Compared with those who did not develop a SPM, pediatric lymphoma survivors with SPM had much worse survival outcomes (Fig. 4B).

### Factors influencing second primary malignancies in patients with pediatric lymphoma

**Fig. 5:**
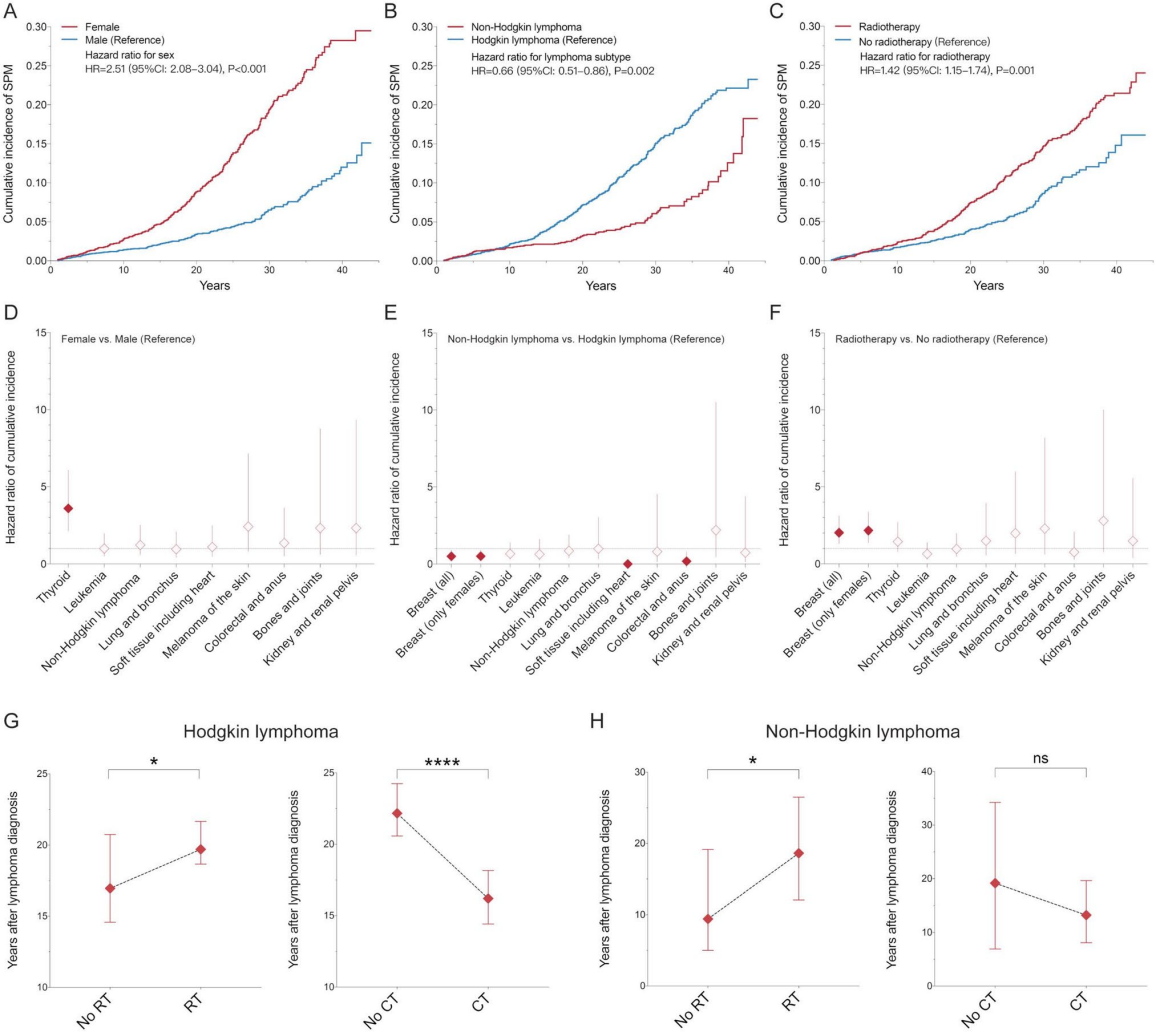
Risk patterns and characteristics of second primary malignancies (SPM) among pediatric lymphoma patients diagnosed between 1975 and 2018 from the SEER database. **A**–**C** Cumulative incidence of SPM by multivariate competing-risk analysis among pediatric lymphoma patients distributed by sex (males as reference), lymphoma subtype (Hodgkin lymphoma as reference) and radiotherapy (RT; no RT as reference). **D**–**F**, hazard ratio (HR) of SPM at the most common sites among pediatric lymphoma patients distributed by sex (males as reference), lymphoma subtype (Hodgkin lymphoma as reference) and RT (no RT as reference). Red diamonds correspond to a significant difference for each SPM site (P < 0.05) by Fine-Gray test in the multivariate competing-risk analysis. **G**, **H** Interval time (years) between lymphoma diagnosis and SPM for different treatments by RT and not receiving RT, and by chemotherapy (CT) and not receiving CT. The results in this figure were analyzed excluding cases (N = 179) with unspecified record in the Case-Listing session of the software SEER*Stat

By competing risk regression, we found that female, HL and receiving RT are associated with a significantly higher risk of SPMs (Fig. 5A–C). Over the past decade, there is limited variation in the incidence of SPMs among different treatment periods (Supplemental Fig. 1). Female survivors, whether of HL or NHL, were more susceptible to SPMs than males (hazard ratio, HR: 2.51, 95% CI 2.08–3.04, P < 0.001). By analyzing SPM sites (Fig. 5D), second primary thyroid cancer showed an elevated 3.59-fold (95% CI 2.12–6.07, P < 0.001) risk in females compared with males. Compared with HL, NHL was associated with a significantly (34%) decreased risk of SPMs (HR: 0.66, 95% CI 0.51–0.86, P = 0.002, Fig. 5B), especially female breast cancer (HR: 0.51, 95% CI 0.28–0.92, P = 0.026), soft tissue malignancies (HR < 0.001), and colorectal and anal cancer (HR: 0.18, 95% CI 0.04–0.80, P = 0.024) (Fig. 5E). It was surprising that 21 patients developed a soft tissue malignancy after HL, while there was none observed after NHL. Moreover, RT did obviously increase the risk of SPMs (HR: 1.42, 95% CI 1.15–1.74, P < 0.001), mainly for some second cancers, such as female breast cancer (HR: 2.16, 95% CI 1.38–3.36, P < 0.001) (Fig. 5F).

In spite of increased risks of SPMs caused by RT, SPMs in RT-treated patients occurred significantly later than those in the non-RT group, it approximately 9.2 and 2.7 years delay for NHL and HL, respectively (Fig. 5G,H**)**: pediatric NHL survivors (RT, median time: 18.6, 95% CI 12.1–26.5 years vs. non-RT: 9.4, 95% CI 5.0–19.2 years, P = 0.0279) and pediatric HL survivors (RT, median: 19.7, 95% CI 18.7–21.7 years vs. non-RT: 17.0, 95% CI 14.6–20.8 years, P = 0.0458). Thus, delayed effects of RT make long-term observation and surveillance particularly necessary. Although CT-treated survivors did not show an incidence increase of SPMs (Supplemental Table 2), CT significantly accelerated the occurrence of SPMs by about 6 years in pediatric HL (CT: 16.2, 95% CI 14.4–18.2 years vs. non-CT: 22.2, 95% CI 20.6–24.3 years, P < 0.0001; Fig. 5G).

## Discussion

Advancements in lymphoma treatments have significantly extended the life expectancy of patients, creating a growing concern, especially for the pediatric population with the emergence of SPMs. This study aimed to delve into the lifetime risks of SPMs after pediatric HL and NHL, utilizing the comprehensive SEER database.

Our findings revealed a remarkable 4.36-fold increase in the risk of SPMs among pediatric lymphoma survivors, underscoring the gravity of this issue. The risk of SPMs varied among subgroups, with sex, age, race, latency since diagnosis, primary lymphoma site, subtype, Ann Arbor stage, and treatment modalities all playing significant roles. Of particular importance was the identification of the highest SIR within the initial 1–5 years following lymphoma diagnosis, emphasizing an early vulnerability window. These results stress the necessity for prolonged surveillance and vigilance among pediatric lymphoma survivors. Notably, distinct histological subtypes of lymphoma carried significantly elevated SPM risks, accentuating the need for a subtype-specific approach to patient care. The associations observed with certain subtypes such as nodular lymphocyte-predominant HL, B-cell precursor NHL, T-cell precursor NHL, and mycosis fungoides underscore the importance of considering this diversity and the potential immune vulnerability within the pediatric lymphoma population (Chihara et al. 2021; Moser et al. 2021).

Furthermore, this research disclosed a substantial and increasing risk of SPMs over a 40-year timeframe, particularly for NHL and HL survivors. Breast and thyroid cancers were the predominant SPMs, highlighting the need for tailored screening and preventative strategies, especially for female patients. Several studies reported a similar incidence of SPMs (about 5%) for pediatric NHL (Bluhm et al. 2008; Friedman et al. 2010; Leung et al. 2001; Moser et al. 2021), but we report here for the first time a much higher 40-year cumulative incidence for both NHL (12.6%) and HL (22.2%), suggesting a lifetime effect.. The increased incidence of SPMs over time emphasizes the severe threat to pediatric lymphoma patients, necessitating more frequent and earlier malignancy screenings throughout their lives. These findings underscore the importance of personalized, lifelong care and screening protocols for this unique patient population to mitigate the impact of SPMs on their health and well-being.

While previous studies on SPMs among pediatric lymphoma survivors have been limited by sample size, this research has quantitatively analyzed influential factors. Gender, lymphoma subtype, and treatment modalities emerged as critical determinants of SPM risk. Female patients, irrespective of lymphoma subtype, exhibited a heightened susceptibility to SPMs, with HL patients at increased risk compared to NHL cases. Notably, the study for the first time demonstrated a 3.59-fold elevated risk of second primary thyroid cancer in females compared to males. These findings underline the significance of individualized care and long-term follow-up for this patient population.

Though previous research has indicated treatment-related SPM risks, few have explored whether CT or RT could accelerate SPM development (Morton et al. 2010; Moser et al. 2021). This study sheds further light on the impacts of treatment modalities on SPM risk. It is noteworthy that while CT did not elevate the overall SPM incidence, it significantly expedited SPM development in pediatric HL cases. This highlights the need for close monitoring and management of patients receiving CT, enabling the early detection of SPMs. The data indicate a correlation between CT and the accelerated development of certain SPMs, especially leukemia. However, it is essential to consider the multifactorial nature of cancer development and the potential influence of treatment modalities. This underscores the importance of close monitoring and management of patients undergoing CT, focusing on early detection of SPMs, particularly in the context of the observed heightened risk in pediatric HL cases, especially in the leukemia category. Furthermore, radiotherapy was linked to a substantial increase in SPM risk, especially for certain secondary cancers like female breast cancer. Importantly, the adverse effects of RT were characterized by a considerable delay, emphasizing the necessity of long-term observation and surveillance.

Nevertheless, it is crucial to acknowledge specific limitations in our study. Most notably, we were unable to provide detailed treatment information, encompassing cumulative doses of chemotherapeutic drugs or radiation. Unfortunately, the SEER database did not furnish detailed information about the location of RT in our study, thereby limiting our ability to explore the relationship between the location of RT and the subsequent occurrence of SPMs. Additionally, our study does not delve into the evolving landscape of treatment strategies over the 40-year period, a facet we recognize as crucial. Furthermore, our investigation did not explore the influence of biological, immunological, or other predisposing factors in the development of SPMs. In addition to these limitations, it is essential to note that the SEER program is a widely used comprehensive cancer registry system in the United States, covering approximately one-third of the population. While we acknowledge that no population-based registry can be entirely exhaustive, the SEER program is designed to be as representative as possible, drawing from diverse geographic regions and population groups. Regarding the quality and completeness of data, the SEER program employs rigorous data collection and quality control measures. We acknowledge any potential limitations in completeness, recognizing the inherent trade-offs in data integrity.

## Conclusions

In conclusion, more attention should be paid to SPM-related signs or symptoms in pediatric lymphoma survivors for early detection and intervention, and that more studies are required to elucidate potential mechanisms of association between pediatric lymphomas and SPMs.

### Supplementary Information

Below is the link to the electronic supplementary material.

Supplementary file1 (PDF 213 KB)

## Data Availability

The data is available on the Surveillance, Epidemiology, and End Results (SEER, http://seer.cancer.gov) database.
