# Supplementary material for: Lifetime risks of second primary malignancies after pediatric Hodgkin lymphoma and non-Hodgkin lymphoma
Source: J Cancer Res Clin Oncol. 2024 Jan 27;150(2):41. doi: 10.1007/s00432-023-05583-4 (PMC10821973; doi:10.1007/s00432-023-05583-4)

Supplemental Table 1. Risk of second primary malignancies (SPM) among pediatric lymphoma patients by specific lymphoma subtypes.

Lymphoma subtype	No. of patients	Observed no. of SPM	Expected no. of SPM	SIR	95%CI	Excess risk (per 10,000)	PY at Risk	Mean PY at Risk	Mean Age at Exposure	Mean Age at SPM
**Total**	6984	478	109.7	4.36#	3.98-4.77	32.9	112058	16.0	14.2	35.1
**1 Hodgkin Lymphoma**	3983	371	78.2	4.74#	4.27-5.25	42.3	69265	17.4	15.8	36.3
**1(a) Classical Hodgkin lymphoma**	3828	360	77.4	4.65#	4.19-5.16	41.8	67668	17.7	15.9	36.4
**1(a)1 Lymphocyte-rich/mixed cell/lymphocyte depleted**	599	57	14.0	4.08#	3.09-5.29	34.2	12575	21.0	14.6	39.1
1(a)1.1 Lymphocyte-rich	144	13	3.3	3.94#	2.10-6.74	30.9	3138	21.8	14.2	34.0
1(a)1.2 Mixed cellularity	429	39	9.9	3.93#	2.80-5.38	32.9	8838	20.6	14.7	39.2
1(a)1.3 Lymphocyte-depleted	26	5	0.8	6.69#	2.16-15.61	70.9	600	23.1	15.6	51.6
**1(a)2 Nodular sclerosis**	2826	270	57.5	4.69#	4.15-5.29	42.8	49674	17.6	16.1	36.2
**1(a)3 Classical Hodgkin lymphoma, NOS**	403	33	5.9	5.63#	3.87-7.91	50.1	5419	13.5	15.8	33.3
**1(b) Nodular lymphocyte predominant Hodgkin lymphoma**	155	11	0.9	13.00#	6.48-23.27	63.6	1598	10.3	14.1	35.0
**2 Non-Hodgkin lymphoma**	2995	107	31.5	3.48#	2.83-4.23	17.7	42781	14.1	12.1	30.7
**2(a) Non-Hodgkin lymphoma, B-cell**	1880	68	22.9	2.97#	2.31-3.77	15.7	28793	15.3	12.5	33.0
**2(a)1 Precursor Non-Hodgkin lymphoma, B-cell**	119	2	0.2	11.17#	1.25-40.32	25.3	720	6.1	8.5	9.2
**2(a)2 Mature Non-Hodgkin lymphoma, B-cell**	1461	50	17.6	2.84#	2.11-3.75	14.4	22447	15.4	12.9	33.2
2(a)2.1 Mantle cell lymphoma	24	4	0.6	6.75#	1.82-17.29	62.5	545	22.7	9.9	52.4
2(a)2.2 Diffuse large B-cell lymphoma (DLBCL)	711	29	10.7	2.72#	1.82-3.91	16.3	11252	15.8	14.7	30.3
2(a)2.3 Burkitt lymphoma/leukemia	568	12	4.6	2.63#	1.36-4.60	8.7	8571	15.1	10.2	31.0
2(a)2.4 Follicular lymphoma	105	5	1.5	3.36#	1.08-7.85	21.8	1611	15.4	14.5	40.4
**2(a)3 Non-Hodgkin lymphoma, B-cell, NOS**	300	16	5.1	3.14#	1.80-5.11	19.4	5626	18.8	11.9	35.1

**2(b) Non-Hodgkin lymphoma, T-cell**	623	15	2.3	6.44#	3.60-10.62	23.4	5414	8.7	12.0	23.8
**2(b)1 Precursor Non-Hodgkin lymphoma, T-cell**	199	3	0.3	9.10#	1.83-26.59	22.0	1213	6.1	10.4	16.0
**2(b)2 Mature Non-Hodgkin lymphoma, T-cell**	424	12	2.0	6.00#	3.10-10.48	23.8	4201	9.9	12.8	25.7
2(b)2.1 Mycosis fungoides	94	5	0.6	9.08#	2.93-21.19	48.4	919	9.8	13.5	28.5
2(b)2.2 Peripheral T-cell lymphoma	324	7	1.4	4.86#	1.95-10.00	17.1	3252	10.0	12.6	23.8
Peripheral T-cell lymphoma, NOS	66	3	0.4	6.91#	1.39-20.19	31.5	813	12.3	11.3	25.9
Anaplastic lar cell lymph, T-/Null-cell	185	3	0.8	3.79	0.76-11.09	11.8	1880	10.2	12.5	22.2
Cutaneous T-cell lymphoma, NOS	27	1	0.1	10.24	0.13-56.97	35.7	252	9.4	14.1	22.3
**2(c) Non-Hodgkin lymphoma, unknown lineage**	330	16	3.3	4.90#	2.80-7.96	22.6	5647	17.1	10.2	27.6
**2(d) Non-Hodgkin lymphoma, NOS**	162	8	3.0	2.64#	1.14-5.20	17.0	2927	18.1	12.6	30.0

SPM, second primary malignancy. SIR, standardized incidence ratio. PY, person-year. NOS, not otherwise specified.

#Significant difference was observed ($P<0.05$).

All results in this table were calculated and analyzed by MP-SIR session of the software SEER*Stat with a 1-year latency of SPM after the first primary lymphoma.

Supplemental Table 2. Multivariate competing-risk analysis of second primary malignancies (SPM) among pediatric lymphoma patients for demographic characteristics.

**Characteristic**	**HR**	**95%CI**		**P value**
		**Lower**	**Upper**	
Sex				
Male	Reference	-	-	
Female	2.51	2.08	3.04	**<0.001**
Age, years				
<1	1.86	0.21	16.60	0.58
1-4	Reference	-	-	-
5-9	0.88	0.45	1.72	0.72
10-14	1.35	0.73	2.48	0.34
15-19	1.54	0.85	2.81	0.16
Race				
White	Reference	-	-	-
Black	1.21	0.89	1.65	0.23
Other†	1.25	0.81	1.92	0.32
Unspecified	NA	NA	NA	NA
Lymphoma subtype				
Hodgkin lymphoma	Reference	-	-	-
Non-Hodgkin lymphoma	0.66	0.51	0.86	**0.002**
Ann Arbor stage				
Stage I	Reference	-	-	-
Stage II	0.78	0.57	1.07	0.12
Stage III	0.92	0.62	1.36	0.68
Stage IV	0.94	0.63	1.38	0.74
Unstaged	NA	NA	NA	NA
Chemotherapy				
No	Reference	-	-	
Yes	0.94	0.77	1.16	0.58
Radiotherapy				
No	Reference	-	-	-
Yes	1.42	1.15	1.74	**0.001**
Unspecified	NA	NA	NA	NA

†Other in race correspond to American Indian/AK Native, Asian/Pacific Islander. The results in this table were calculated and analyzed by R software with package “cmprsk” and “survival”.

Supplemental Figure 1. Cumulative incidence of SPM by multivariate competing-risk analysis among pediatric lymphoma patients distributed by year of diagnosis. A. Follow up of 10 years; B. Follow up of 20 years; C. Follow up of 30 years

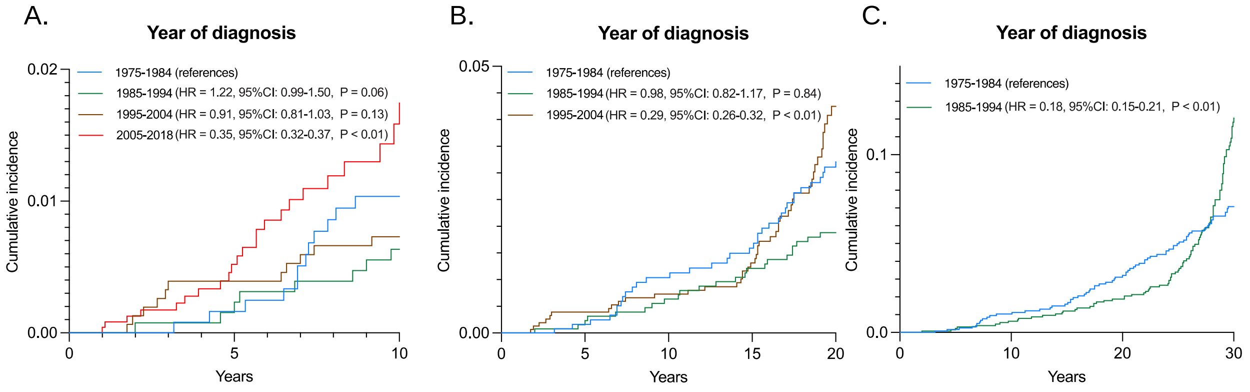

Supplement: Supplementary file 1 — Supplementary file1 (PDF 213 KB) [file 432_2023_5583_MOESM1_ESM.pdf]
